# Prevention of Inflammation, Neovascularization, and Retinal Dysfunction by Kinin B_1_ Receptor Antagonism in a Mouse Model of Age-Related Macular Degeneration

**DOI:** 10.3390/jcm12196213

**Published:** 2023-09-26

**Authors:** Menakshi Bhat, Shima Shirzad, Abdel-Rahamane Kader Fofana, Fernand Gobeil, Réjean Couture, Elvire Vaucher

**Affiliations:** 1 School of Optometry, Université de Montréal, Montreal, QC H3T 1P1, Canada; menakshi.bhat@umontreal.ca (M.B.);; 2 Department of Pharmacology and Physiology, Faculty of Medicine, Université de Montréal, Montreal, QC H3T 1J4, Canada; rejean.couture@umontreal.ca; 3 Department of Pharmacology and Physiology, Faculty of Medicine and Health Sciences, Université de Sherbrooke, Sherbrooke, QC J1H 5N4, Canada; fernand.junior.gobeil@usherbrooke.ca

**Keywords:** age-related macular degeneration, kallikrein-kinin system, kinin B_1_ receptor, electroretinography, neovascularization, inflammation, retina, choroid

## Abstract

The kallikrein-kinin system (KKS) contributes to vascular inflammation and neovascularization in age-related macular degeneration (AMD), particularly via the kinin B_1_ receptor (B_1_R). The aim of the present study was to determine the protective effects of the topical administration of the B_1_R antagonist (R-954) on inflammation, neovascularization, and retinal dysfunction in a murine model of neovascular AMD. Choroidal neovascularization (CNV) was induced in C57BL6 mice using an argon laser. A treatment with ocular drops of R-954 (100 μg/15 μL, twice daily in both eyes), or vehicle, was started immediately on day 0, for 7, 14, or 21 days. CNV, invasive microglia, and B_1_R immunoreactive glial cells, as well as electroretinography alterations, were observed within the retina and choroid of the CNV group but not in the control group. The staining of B_1_R was abolished by R-954 treatment as well as the proliferation of microglia. R-954 treatment prevented the CNV development (volume: 20 ± 2 vs. 152 ± 5 × 10^4^ µm^3^ in R-954 vs. saline treatment). R-954 also significantly decreased photoreceptor and bipolar cell dysfunction (a-wave amplitude: −47 ± 20 vs. −34 ± 14 µV and b-wave amplitude: 101 ± 27 vs. 64 ± 17 µV in R-954 vs. saline treatment, day 7) as well as angiogenesis tufts in the retina. These results suggest that self-administration of R-954 by eye-drop treatment could be a promising therapy in AMD to preserve retinal health and vision.

## 1. Introduction

Age-related macular degeneration (AMD) is an elderly disease featuring the degeneration of the retina causing significant central visual impairment. It is the leading cause of visual disability in the industrialized world, with projected cases estimated to be 288 million by 2040 [1]. The process is a combination of non-neovascular drusen and retinal pigment epithelium (RPE) abnormalities (dry AMD), and additional neovascular derangement, i.e., choroidal neovascular membrane formation (wet or exudative AMD) [2,3]. Though dry AMD is the most common subtype, wet AMD is mostly responsible for the cases with severe vision loss [4,5]. The wet form of AMD is characterized by the ingrowth of new and immature vessels, which invade the outer retina from the underlying choroid. In addition, oxidative stress, inflammation, and lipofuscin/drusen accumulation, further reduces the flow of nutrients across the RPE [6]. This choroidal neovascularization (CNV) creates the obstruction of light paths and RPE cell death leading to the loss of photoreceptors and retinal detachment causing permanent vision loss in some patients [1,4,5].

The development of CNV during AMD has been shown in association with many inflammatory factors [7,8]. Amongst them, kinin B_1_ receptor (B_1_R) is a very interesting player due to its involvement in various pathological processes, including inflammation, angiogenesis, vasomotricity, blood-retinal barrier permeability, and pain [9,10]. B_1_R is weakly detectable under normal physiologic conditions while it is strongly expressed in pathologic and inflammatory states, such as Alzheimer’s disease, cardiovascular and renal diseases, infectious diseases, arthritis, and diabetes [11,12,13,14]. In our previous studies, we were able to show that the kallikrein–kinin system (KKS) and B_1_R play a prominent role in retinal damage during diabetic retinopathy and CNV [9,15,16,17]. In one of these studies, we have demonstrated that B_1_R is highly expressed in human wet AMD where it was colocalized with various inflammatory (iNOS, microglia) and fibrotic markers, supporting the proinflammatory role of B_1_R in AMD [17]. One clinical study has successfully ameliorated visual acuity with the use of a plasma kallikrein inhibitor (KVD001) in diabetic macular oedema without causing any retinal thickening or exacerbating the pathology [18]. This further supports a key role for the KKS in retinal disorders.

**Figure 1:**
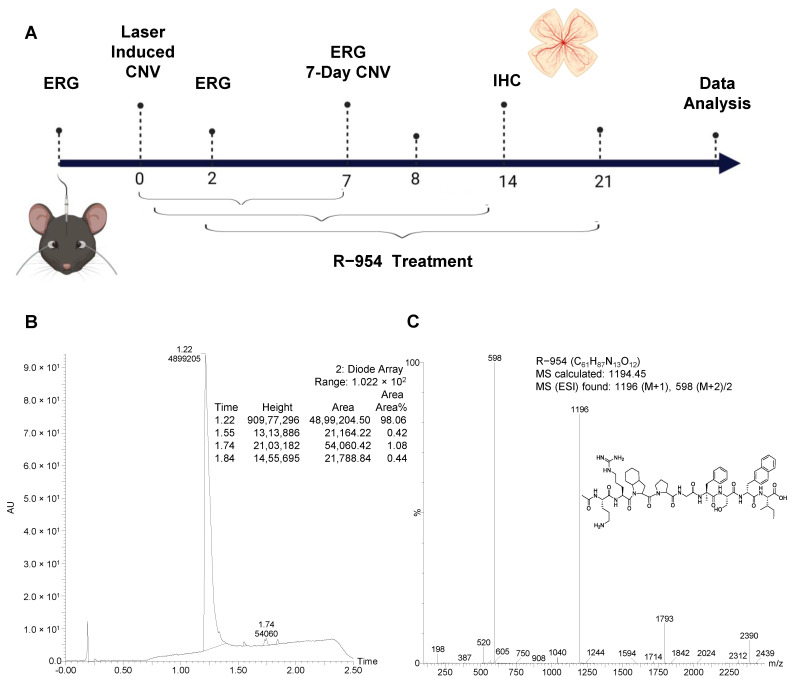
Schematic representation of experimental design of the study. Timeline of the study (**A**): the R-954 treatment was started immediately after CNV induction on day 0, and continued for 7-, 14- or 21-days in three different experimental groups. ERG was performed before CNV and during the treatment on day 2 and day 7. Choroidal and retinal tissues were collected at the end of the study for immunohistochemistry and confocal microscopy. HPLC and mass spectrometry analyses show mass confirmation and purity (98%) of the synthesized peptide B_1_R antagonist R-954 (**B**,**C**). The molecular formula and mass spectrum (MS) calculated of R-954 are indicated (**C**). CNV, choroidal neovascularization; ERG, electroretinogram; IHC, immunohistochemistry; R-954, B_1_R antagonist; MS, mass spectrometry; ESI-MS, Electrospray ionization (positive mode)-mass spectrometry; *m/z*, mass-to-charge ratio.

Based on the above reports, the objective of the present study was to assess the effects of the B_1_R antagonist R-954 in the prevention of inflammation, neovascularization, and angiogenesis in the laser-induced CNV in a murine model of AMD (Figure 1).

## 2. Materials and Methods

### 2.1. Animals

All experimental methods and animal care procedures were approved by the animal care committee of Université de Montréal (CDEA, protocol No. 21-035, approved 31 March 2021; and protocol No. 22-044), in compliance with the guiding principles for animal experimentation as enunciated by the Canadian Council on Animal Care and ARVO statement for use of animals (The Association for Research in Vision and Ophthalmology—Statement for the Use of Animals in Ophthalmic and Vision Research, arvo.org). Food and water were supplied ad libitum and the animals were kept in a 12 h light/dark cycle and in a temperature-controlled room (22 ± 2 °C). From the in-house colony of Université de Montréal, thirty-eight C57BL6 mice (6–8 weeks old, 23 males: 27 ± 4 g and 15 females 22 ± 2 g) were employed for the study. For the 7-day time period, animals were separated into three groups: sham, CNV + saline and CNV + R-954. For each CNV animal, one eye received the laser shot and the other did not and was taken as a treatment control (n = 8 mice/group). For the 14-day (n = 3/group) and 21-day (n = 4/group) time period, the sham group was omitted.

### 2.2. Induction of Experimental CNV

Mice were anaesthetized with an intraperitoneal injection of a mixture of 80 mg/kg ketamine (Narketan, 100 mg/mL; Vetaquinol, Towcester, UK) and 2 mg/kg xylazine (Xylamax, 100 mg/mL; Bimeda, Cambridge, ON, Canada). Both corneas were locally anesthetized using 0.5% proparacaine hydrochloride solution (Alcaine^®^, Alcon, QC, Canada), pupils were dilated using 2.5% phenylephrine (Mydfrin^®^, Alcon, QC, Canada) and ophthalmic liquid gel (Tear-Gel, Bausch & Lomb, Guelph, ON, Canada) solution drops were applied to prevent eye dryness. The CNV was performed based on the method previously described by Gong et al. in 2015 and adapted by Hachana et al. in 2020 [16,19]. The mouse was positioned in dorsal decubitus on a heated stage and was monitored to maintain a core body temperature of 37 °C. Laser photocoagulation was performed through a slit lamp argon-green laser with a wavelength of 532 nm (Coherent Novus 2000; Carl Zeiss, Oberkochen, Germany) guided by a target beam. A 90-diopter lens was used to view the fundus of the eye. Five laser burns (0.1 s duration, 110 mW and 100 μm diameter spot) were induced around the optic nerve in the right eye, while the left eye served as the control. Laser burns were given carefully to avoid large vessels, but as close as possible to the optic nerve head (around 500 µm, see Section 3). Laser-induced disruption of Bruch’s membrane was identified by the appearance of a bubble at the site of laser lesion [19,20,21]. In 16% of the cases, the laser spot was, however, localized more peripherally due to the anatomy of the vascular bed. At the end of the intervention, 2.25 mg/kg atipamezole (Revertor, 5 mg/mL; Modern Veterinary Therapeutics, Miami, FL, USA) was injected to reverse the effect of sedation.

### 2.3. Topical Administration of B_1_R Antagonist R-954

Pharmacological blockade of the B_1_R was achieved with a highly selective and metabolically stable peptide antagonist R-954 provided by Dr. Gobeil Jr, Université de Sherbrooke, Canada [22]. The peptide R-954 was assembled on solid support by an automated Pioneer peptide synthesizer using Fmoc (9-fluorenylmethyoxy-carbonyl) chemistry. The peptide was purified by reverse phase HPLC on Waters 2535 module (with Waters 2489 UV detector) with a preparative ACME C18 (10 μm, 250 × 30 mm) column. The pure product fractions were pooled, lyophilized, and stored at −20 °C. Compound purity and identification were assessed by UPLC-UV-Mass spectroscopy (Waters Aquity H-Class, SQD2 (ESI) mass detector) with a Waters BEH C18 (1.7 μm, 2.1 × 50 mm) column. According to UPLC-MS analysis, the purity of the peptide R-954 exceeded 98%, with expected mass spectra (Figure 1).

Animals received 100 μg/15 μL of R-954 topically by eye drops, immediately after the laser burn on day 0 and twice daily for 7-, 14- or 21-days in both CNV and non-CNV eyes. Controls received 15 μL of vehicle (saline) twice daily in both CNV and non-CNV eyes.

### 2.4. Electroretinogram Recordings

**Figure 2:**
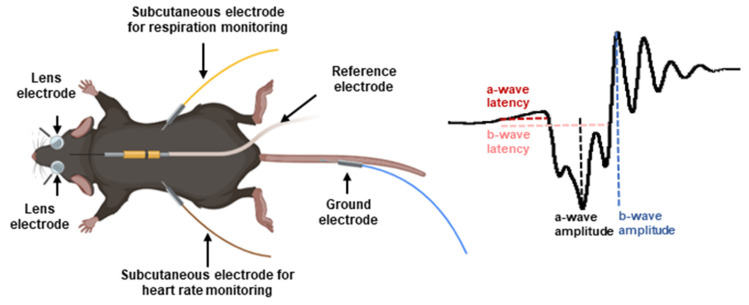
Representation of an anesthetized mouse placed under a Ganzfeld dome with photo stimulator while performing electroretinography. The electrode was positioned on a corneal lens adapted for mice. A reference electrode was located subcutaneously in the forehead and ground electrode in the tail of the mouse. Peak amplitude and latency of a- and b-waves were measured as shown on the schematic representation of an electroretinogram trace (right panel).

Animals were dark-adapted for 12 h, and all the experiments were carried out under dim red illumination light. ERG was performed using protocol adapted by Cecyre et al., 2020 [23], on anaesthetized mice using isoflurane inhalation (1.5%). The corneas were anesthetized with a drop of 0.5% proparacaine hydrochloride and pupils were dilated with a drop of 2.5% phenylephrine. The mice were then positioned on a probed heating pad held at 37 °C under a Ganzfeld dome that housed a photo stimulator. Heart rate and respiration were monitored through subdermal electrodes linked to a sound amplifier. The ERGs were recorded with a gold electrode placed on a corneal lens adapted for mice (LKC Technologies, Gaithersburg, MA, USA) (Figure 2). A drop of eye lubricant (Systane Ultra, Alcon, Canada) was used while placing an electrode on the mouse eye. Reference and ground electrodes (E2 subdermal electrode; Grass Instruments) were placed subcutaneously in the forehead and in the tail. ERG performed under scotopic conditions consisted of 5 flashes (luminance from −3.89 to 1.40 log scot·cd·s·m^−2^, 30 s interstimulus interval). Signals were amplified (bandwidth 1–1000 Hz; 10,000×; P511, Astro-med, Inc, Grass Instrument, West Warwick, RI, USA) and acquired with an acquisition system (1401, CED, Cambridge, UK) to generate broadband ERGs using the software Signal (v.3.01x, CED, Cambridge, UK). Both eyes were recorded independently. Pre CNV-ERG was performed on naïve control mice prior to CNV lesion to obtain the ERG baseline. ERG was repeated after photocoagulation on day 2 and day 7. All analyses were carried out by an experienced observer blind to experimental conditions.

### 2.5. Electroretinogram Analysis

ERGs are composed of electrical potentials identified as a-wave, b-wave, and oscillatory potentials (Ops). Analysis of the a- and b-waves was performed using a 40 Hz low pass digital filter and eliminating the noise from Ops. Peak a-wave amplitude was measured from baseline to the initial negative-going voltage, whereas peak b-wave amplitude was measured from the trough of the a-wave to the peak of the positive b-wave of the retinal response (Figure 2). Implicit times were measured from flash onset to the peak of the waves. Ops were also analysed similarly, using a 60 Hz high pass digital filter to eliminate the a- and b-waves. The amplitude of Ops was measured and reported individually and as the sum of all Ops.

### 2.6. Flat Mount Dissection and Immunofluorescent Staining

Experimental mice were humanely euthanized with pentobarbital one day after the 7-day ERG and at 14-day or 21-day. Eyes were enucleated and fixed in freshly prepared 4% paraformaldehyde (Sigma-Aldrich, Oakville, ON, Canada) in phosphate buffer solution (PBS) for 1 h at room temperature (RT) as described previously [15]. Eyes were dissected to retrieve wholemount retina and choroid under stereo microscope (Mz95, Leica Microsystems, Buffalo Grove, IL, USA). Wholemount choroid and retina were incubated for 1 h at RT in PBS containing 2% donkey serum (Jackson ImmunoResearch Laboratories Inc, London, ON, Canada) to block the non-specific binding sites and 0.25% triton X-100 (Thermo Fisher Scientific, Mississauga, ON, Canada) to permeabilize the membranes. The goal of the immunostaining was to determine: (1) the neovascular alteration, (2) reactive micro and macroglia, (3) if B_1_R was overexpressed in CNV eye, indicative of inflammation, and by which cells, and if the staining was reduced by R-954 treatment. The polyclonal rabbit antibody for B_1_R has been characterized and tested by immunohistochemistry and western blot using knockout mice and siRNA approaches in previous studies [11,15]. Tissues were incubated overnight at 4 °C with a mixture of the following primary antibodies: 1:750, polyclonal rabbit anti-B_1_R; 1:10, isolectin GS-IB4 coupled to Alexa Fluor 568 (I21412; Thermofisher Scientific, Mississauga, ON, Canada) for staining endothelial cells; and 1:2000, rat monoclonal anti-Iba-1 (ab283346 Abcam, Toronto, ON, Canada) to stain microglia. Sections were further incubated at RT for 2 h with Alexa Fluor 488 donkey secondary anti-rabbit IgG (A21206, Invitrogen, Burlington, ON, Canada) to visualize B_1_R and Alexa Fluor 647 goat anti-rat IgG (A21247, Invitrogen, Burlington, ON, Canada) to identify Iba-1 positive microglial cells.

B_1_R was clearly expressed by microglial cells in saline treated CNV eyes. To determine if B_1_R was expressed by Müller cells or astrocytes, saline treated whole mounted retinas were blocked with 2% donkey serum in PBS for 2 h and incubated overnight with 1:500 mouse monoclonal anti-glial fibrillary acid protein GFAP (ab4674; Abcam, Canada) to label astrocytes or Müller cells and 1:200 guinea-pig monoclonal anti-Kir4.1 (KCNJ10) antibody (AGP-012, Alomone Labs, Jerusalem, Israel) to recognize inward rectifier Kir4.1 potassium channels overexpressed by activated Müller cells of the retina. Retinas were flat mounted with the photoreceptor layer facing up and preserved using antifade mounting medium (ProLong Gold; Invitrogen).

Microphotographs were taken using FV3000 Olympus laser scanning confocal microscope (FV3000 Confocal Laser Scanning Microscope, Evident Canada, Quebec, QC, Canada). High magnification photographs (60× or 40×, oil immersion) were taken at random and by targeting every CNV when present. No correspondence between choroid and retina damage could be performed, as choroid and retina were dissected separately. For some representative choroids or retina, a map reconstruction was performed from thirty-five photographs—12 h scans—using a 10× objective with automatic scanning and the stitching function. The microphotographs were deconvolved to max intensity using cellSense Imaging Software (Software cellSens Standard Version 4.1 CS-ST-V4.1).

### 2.7. Quantitative Measurement of Choroidal Neovascularization and Inflammation

Images were analysed using the open-source software *Image*J developed by the National Institutes of Health (NIH), Bethesda, MD, USA. For quantification of immunostaining, CNV areas or random photographs (sham group) in choroid and retina stained with isolectin GS-IB4, B_1_R and Iba1 were quantified. All the RGB images were divided into red, green, and blue channels, representing isolectin GS-IB4, B_1_R, and microglia staining, respectively. Images were converted into grey levels (8-bit images) and thresholding was applied to differentiate the regions of interest from the background within the choroid or retina. The values were reported as mean ± SEM of total area of fluorescent (grey) pixels in arbitrary units (AU) of isolectin GS-IB4, B_1_R and microglia staining (10 photographs per group, Table 1).

For CNV volume and area quantification in the choroid, images were converted to greyscale with 255 level of intensity (8-bit image). This was carried out to enhance the contrast and visibility of CNV in the choroidal images. Relative CNV volume was determined on high magnification photographs in wholemount choroid stained with A568-isolectin GS-IB4. Z stack reconstructed CNV from confocal microscopy microphotographs were analysed using ImageJ macro code to count the number of laser spots showing CNV and neovessels clusters not associated with laser spots and measure the total area and volume of CNV of each rupture site (ImageJ Visikol^®^—Blog Post: Loading and Measurement of Volumes in 3D Confocal Image Stacks with ImageJ Visikol). An established and constant threshold was used for quantification. The Z stacks of images were loaded directly on the ImageJ and converted to hyperstack. The calibration information, the resolution defined by the image acquisition parameters, was input for each z-stack which ensured there was no distortion of the images. To measure volumes or to repeat measurements across the entire stack, the channels were split, and thresholding of the images was performed. Parameters for measurements were used on the threshold images. Once the measurements were configured macro code (Visikol) was used to measure volume. The macro will loop through each image in the stack and take the measurement of CNV area for each slice individually. At the end, macro code loop through the images, sum the area measurement and multiply this sum by the depth of each slice, displaying the volume of CNV for each image.

### 2.8. Statistical Analysis

Data are presented as mean ± SD/SEM. The group size selection is kept constant for all the experimental groups on which statistical analyses were conducted. Normal (Gaussian) distribution of the data was confirmed using both visual Q-Q plots and the Shapiro-Wilk test. A factorial 5 × 8 (group × flash intensity) ANOVA was performed to analyse the differences in ERG amplitudes and latencies between groups (n = 8 per group, 7-day treatment only) [23,24]. One-way ANOVA was used for comparing area, volume, or quantification of immunostaining, separately, on at least 10 independent measurements (area, volume of CNV or total area stained) within groups [25]. All post-hoc comparisons were conducted using Bonferroni correction. For parametric variables, post-hoc comparisons were conducted only if the main effect was statistically significant, and there was no significant variance in homogeneity. We took care highlighting every possible comparison between the study groups. All outliers were included during data analysis and presentation. Statistical analysis was performed using the Prism™ version 9.5.1 (GraphPad Prism, RRID:SCR_002798, GraphPad Software Inc., La Jolla, CA, USA). The level of statistical significance (*p*) value was set at *p* < 0.05. All data collection was performed by experimenters blinded to treatment groups.

## 3. Results

### 3.1. Development of Neovascularization in the Murine Model of AMD

**Figure 3:**
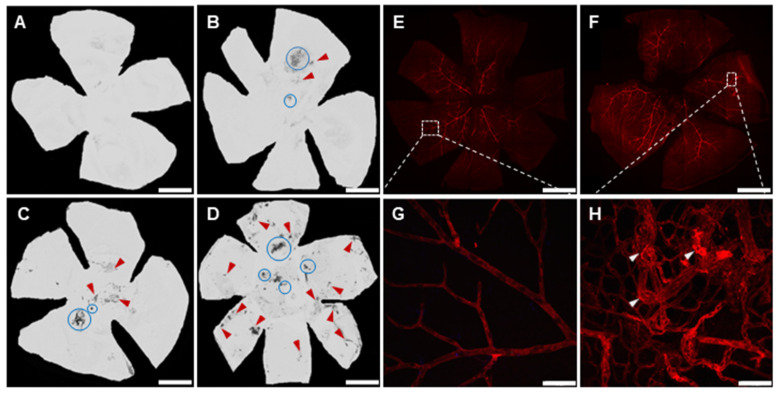
The representative wholemount choroidal maps of non-CNV contralateral control (**A**), and 7-day (**B**), 14-day (**C**) and 21-day (**D**) post CNV, with visible CNV laser spots (circle) and neovessels clusters (red arrowheads). Retinal maps of non-CNV contralateral control (**E**) exhibit well defined vasculature and 21-day post CNV (**F**) demonstrating tangled neovessels formation (white arrowheads). (**G**,**H**) are the high magnification images of dashed boxes in (**E**,**F**), respectively. Scale bar 1 mm (**A**–**F**) and 50 µm (**G**,**H**).

**Figure 4:**
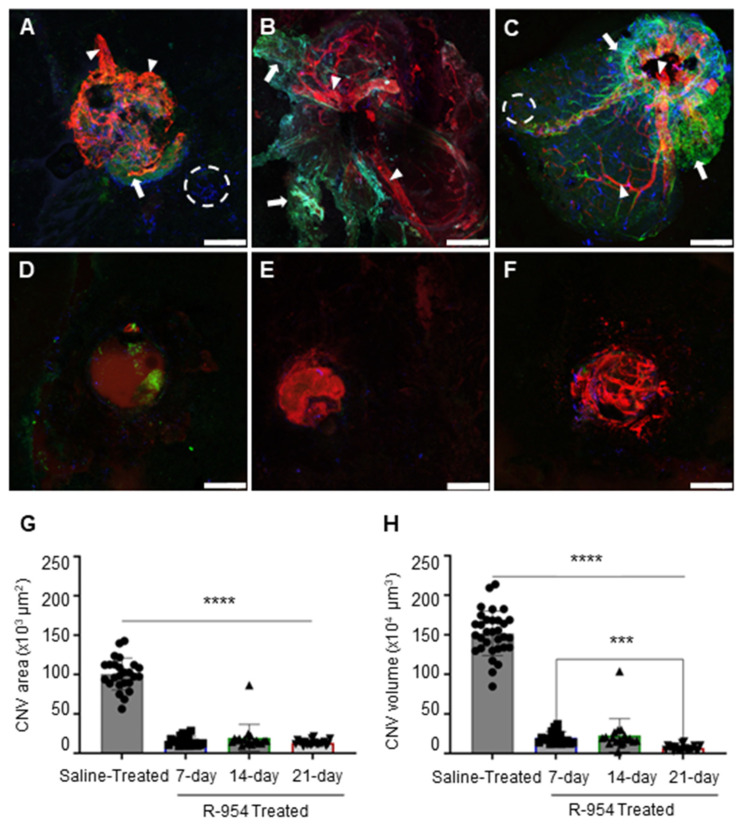
Effect of R-954 treatment on CNV size. Representative photomicrographs of 7-day (**A**), 14-day (**B**) and 21-day (**C**) saline treated CNV choroids and 7-day (**D**), 14-day (**E**) and 21-day (**F**) R-954 treated CNV choroids. The whole choroids were triple immunolabelled using antibodies against isolectin GS-IB4 (red; arrowhead), B1R (green; arrow) and Iba-1 positive microglial cells (blue; circle). Scale bar 50 µm. Relative measurement of CNV area (**G**) and volume (**H**), calculated from Z-stacks with imageJ. The data were presented as mean ± SD (n = 18–25 CNV/group) for saline treated (filled circle), 7-day R-954 treated (filled square), 14-day R-954 treated (filled triangle) and 21-day R-954 treated (filled triangle pointing down). Statistical analysis was performed using one-way ANOVA followed by Bonferroni test. **** (*p* < 0.0001); *** (*p* = 0.0002), all possible comparisons were shown.

Wholemount choroidal and retinal maps, reconstructed using confocal microscopy, were investigated to assess the development of CNV. Two forms of neovascularization were observed, either directly on the site of laser spots on the choroid or seen away from the laser spot (identified as neovessels clusters). No neovascularization nuclei were observed in choroids of non-CNV contralateral control eyes (Figure 3A). The CNV laser spots were located at 478 ± 193 µm from the optic nerve head (from 300 to 700 µm; 7-day group, from 250 to 700 µm; 14-day group and from 300 to 700 µm; 21-day group). The number of laser spots developing CNV per choroid (2 ± 1; 2 ± 1 and 4 ± 1, for 7-, 14- and 21-day respectively) as well as the number of neovessels clusters not associated with laser spots (1 ± 0.5, 1 ± 1, and 14 ± 5, for 7-, 14- and 21-day respectively) was considerably increased with time (Figure 3A–D), suggesting that neovascularization developed with inflammation. The associated glial reactivity was stronger and more widespread with time, clearly embedding the CNV area (Figure 4).

The robust, consistent, and uniform labelling of retinal vessels were observed within retinas of the CNV group compared to non-CNV contralateral controls indicating increased neo-vessels formation (Figure 3E,F). The retina of 21-day CNV group displayed multiple neovascular branches (Figure 3F,H) compared to retinas of non-CNV contralateral control group with regular vasculature extending from optic nerve to peripheral region (Figure 3E,G). The high magnification microphotographs strongly indicate a clear delineation of abnormal vascular structures and multiple layers of retinal blood vessels in 21-day CNV retina (Figure 3H) compared to non-CNV retina (Figure 3G).

**Table 1:** Quantification of the fluorescent immunostaining in saline-treated and R-954 -treated CNV choroid and retina. Each value represents mean ± SEM of total area of fluorescent staining of images, arbitrary units (AU) (n = 10 per group). * *p* ≤ 0.001, one-way ANOVA.

	Choroid						Retina					
	Saline Treated CNV			R-954 Treated CNV			Saline Treated CNV			R-954 Treated CNV		
***Isolectin GS-IB4***												
7-day	65	±	4	13	±	1 *	151	±	7	34	±	3 *
14-day	162	±	13	26	±	2 *	77	±	2	17	±	3 *
21-day	248	±	12	30	±	4 *	157	±	22	31	±	5 *
***B1R***												
7-day	39	±	2	13	±	1 *	204	±	4	23	±	1 *
14-day	95	±	4	8	±	1 *	217	±	15	13	±	1 *
21-day	172	±	10	14	±	1 *	255	±	18	24	±	2 *
***Iba1***												
7-day	51	±	5	15	±	1 *	133	±	4	11	±	2 *
14-day	82	±	4	3	±	1 *	168	±	20	11	±	1 *
21-day	33	±	16	20	±	1 *	241	±	25	9	±	1 *

### 3.2. R-954 Inhibits Choroidal Neovascularization

The pharmacological effects of R-954 on the development of CNV and the suppression of inflammatory markers (B_1_R and microglia) were evaluated using immunohistochemical analysis. The saline treated choroids clearly display larger CNV areas compared to the R-954 treated choroids (Figure 4). There was a marked increase in Iba-1–positive microglial cell population and B_1_R was expressed in morphologically-identified macroglial cells within saline-treated CNV choroids. A noticeable overexpression of B_1_R was observed in all three saline-treated CNV choroids (Figure 4A–C); however, R-954 treatment demonstrated a successful prevention of the overexpression of B_1_R (B_1_R staining: 39 ± 2 vs. 13 ± 1; 95 ± 4 vs. 8 ± 1 and 172 ± 10 vs. 14 ± 1 AU for 7-, 14- and 21-day of saline- vs. R-954 -treated CNV choroids, respectively; Table 1). Microglial cell population was moderately detected in the outer layer of 7-day saline-treated CNV choroid but was tremendously present in the 14- and 21-day saline-treated CNV choroids (Figure 4A–C), the majority of microglia identified were activated morphology, although some ramified and ameboid microglial cells were also observed. R-954 treatment maintained reduced microglial cell population in CNV choroids (Figure 4D–F, Table 1, Iba1 staining: 51 ± 5 vs. 15 ± 1; 82 ± 4 vs. 3 ± 1 and 33 ± 16 vs. 20 ± 1 AU for 7-, 14- and 21-day of saline vs. R-954 treated CNV choroids, respectively; *p* < 0.0001). Saline treated CNV choroids also exhibited noticeably increased neovascular formation (Figure 4A–C), whereas R-954 treatment demonstrated visible reduction in the tentacular neovascular areas and extent of isolectin GS-IB4 staining (64 ± 4 vs. 13 ± 1; 162 ± 13 vs. 26 ± 2 and 248 ± 12 vs. 30 ± 4 AU for 7-, 14- and 21-day of saline vs. R-954 treated CNV choroids, respectively; Table 1). The effective inhibition of CNV development using R-954 treatment was validated by a significant 4-fold decrease in the CNV area (13 ± 1 × 10^3^ µm^2^) compared to saline-treated CNV group (100 ± 4 × 10^3^ µm^2^) (Figure 4G). In addition, a 10-fold substantial decrease in the CNV volume was observed in 21-day R-954 treated CNV choroids (7 ± 1 × 10^4^ µm^3^) compared to saline-treated CNV choroids (152 ± 5 × 10^4^ µm^3^) (*p* < 0.0001). No statistical differences were observed within CNV areas between R-954 treatment groups (7, 14, and 21-day). Nevertheless, there was a 4-fold decrease in the volume of 21-day R-954 -treated CNV choroids vs. 7-day R-954 -treated CNV choroids (7 ± 1 vs. 20 ± 2 × 10^4^ µm^3^) (*p* = 0.002), suggesting an increasing effect of R-954 treatment over time.

### 3.3. R-954 Prevents Retinal Vascular Bed Alteration

**Figure 5:**
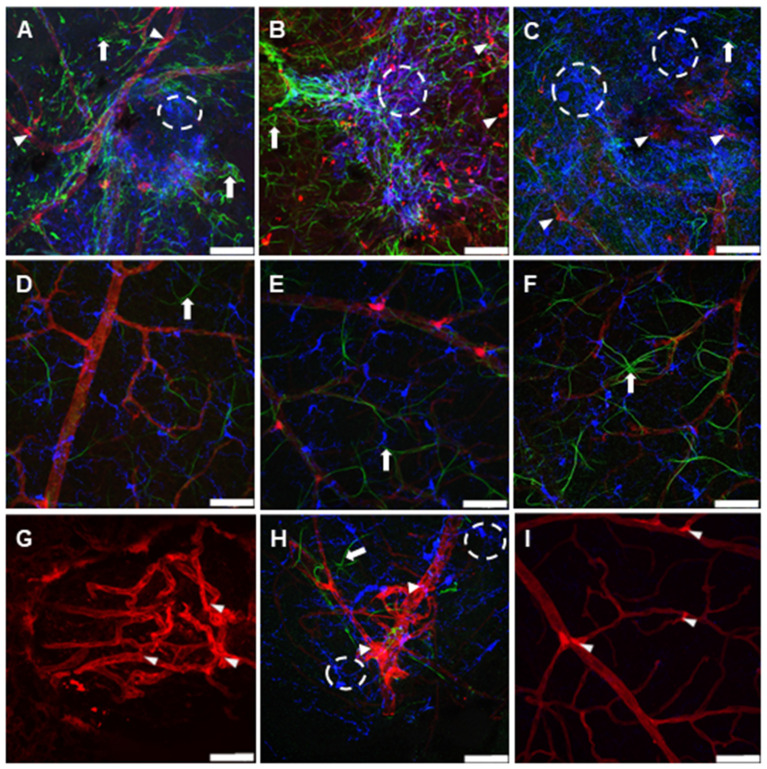
Representative photomicrographs of triple immunolabeled wholemount retinas. Retinas are stained with isolectin GS-IB4 for vascular endothelial cells (red, arrowhead); B_1_R (green, arrow) and Iba-1 positive microglial cells (blue, circle). Upper panels: Retinas from 7-day (**A**), 14-day (**B**) and 21-day (**C**) saline treated CNV eyes. Middle panels: Retinas from 7-day (**D**), 14-day (**E**) and 21-day (**F**) R-954 treated CNV eyes. Lower panels: Magnified images of 21-day saline treated CNV retinas (**G**,**H**) depicting neovascularized lesion stained with isolectin GS-IB4 (**G**); disoriented and dilated blood capillaries and vascular sprouts stained (**H**) or active angiogenesis and normal vasculature in 21-day R-954 treated CNV retina (**I**). Scale bar (**A**–**F**) (50 µm) and (**G**–**I**) (20 μm).

In addition to evaluating the inhibitory effect of R-954 in CNV choroid, its preventive effect on CNV-induced retinal alterations was determined. The development of new blood vessels, enhanced expression of B_1_R and increased microglial population was clearly observed in saline treated CNV retinas after 7-, 14- and 21-days (Figure 5A–C). B_1_R staining increase in saline treated CNV retinas was significantly reduced with R-954 treatment (204 ± 4 vs. 23 ± 1; 217 ±15 vs. 13 ± 1 and 255 ± 18 vs. 24 ± 2 AU for 7-, 14- and 21-day saline vs. R-654 treated CNV retinas, respectively, Table 1). Increased microglial cell activity was seen within all the saline treated CNV retinal groups (Figure 5A–C); however, R-954 treated retinas show reduced microglial population (Figure 5D–F) and successful abolishment of Iba1 staining within 7-, 14- and 21-day R-954 treated retinas (Table 1). Irregular neovascular complexes were tremendously seen within saline treated retinas (Figure 5A–C) and confirmed with strong isolectin GS-IB4 staining (151 ± 7; 77 ± 2 and 157 ± 22 AU for 7-, 14- and 21-day saline treated retinas, Table 1). In comparison, R-954 treatment successfully inhibited neo-vessel formation within 7-, 14- and 21-day treated retinas (Figure 5D–F; Table 1). The vascular sprouts and proliferation (active angiogenesis), and abnormally dilated vessels were clearly visible in 21-day saline-treated CNV retina (Figure 5G,H), whereas R-954 treatment retained normal vascular structure with fine radial branching pattern (Figure 5D–F,I). B_1_R was occasionally present after R-954 treatment; however, microglia population was decreased tremendously indicating normalization of retinal structure. Importantly, R-954 treatment also maintained normal vascular regeneration as active sprouting angiogenesis was seen with typical sprouts at the vascular front (Figure 5I).

### 3.4. Cellular Localization of B_1_R in CNV Retina

**Figure 6:**
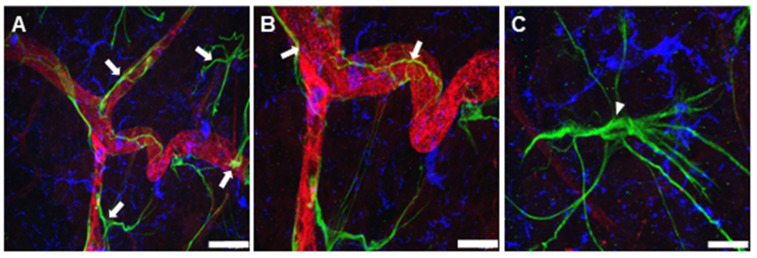
Representative microphotographs demonstrating cellular localization of B_1_R in 21-day saline treated CNV retinas. Retinas were immunostained with isolectin GS-IB4 for vascular endothelial cells (red); B_1_R (green) and Iba-1 positive microglial cells (blue). B_1_R attaches to retinal blood vessels in CNV retina ((**A**,**B**); arrows). Magnified image of B_1_R resembling astrocyte-like cell body ((**C**); arrowhead). Scale bar 20, 10 & 5 µm for (**A**–**C**) respectively.

To understand the morphology and cellular expression of B_1_R, high magnification confocal imaging was performed on the retinas. The presence of B_1_R-stained elements touching the retinal vascular plexus was observed. There was a clear association between B_1_R-labelled filopodia-like structures and blood vessels (Figure 6A,B), including astrocytic or Müller cells endfeet-like patterns. B_1_R labelling was seen on astrocytic cell bodies, featured by flattened cells and a series of fibrous radiating astrocytic endfeet (Figure 6C). Microglial cells were abundantly present in the CNV retina, however there was no co-expression of B_1_R and Iba-1 positive microglial cells (Figure 6C). Also, the microglial cells were not labelled by the lectin GS-IB4, although this lectin does sometimes label reactive microglia.

**Figure 7:**
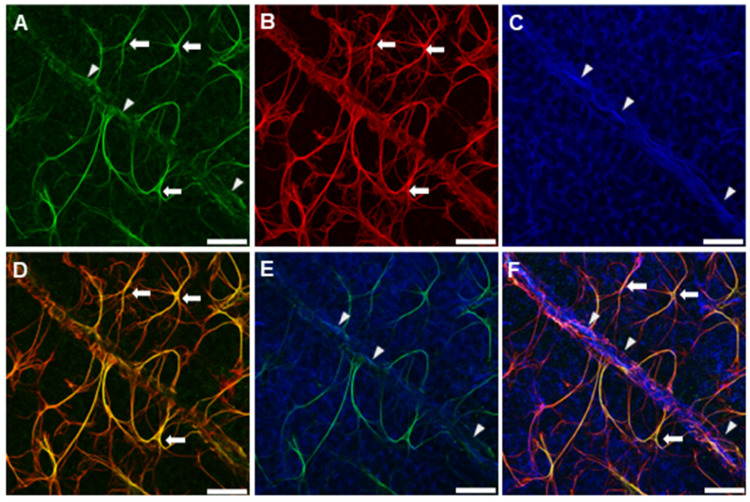
Triple immunostaining of wholemount 21-day saline treated CNV retina with anti-B_1_R antibody (green; (**A**)); anti-GFAP antibody staining astrocytes and activated Müller cells (red; (**B**)) and anti-Kir4.1 antibody preferentially staining Müller cells (blue; (**C**)). An overlay image demonstrating colocalization of B_1_R and GFAP, showing strong localization of B_1_R on glial cells (yellow; arrow; (**D**)). Image indicating co-expression of B_1_R and Müller cells staining Kir4.1 (white; arrowhead; (**E**)). Merged image representing colocalization between B_1_R, GFAP and Kir4.1 (**F**). Scale bar 20 µm.

After observing the resemblance of B_1_R with an astrocyte-like cell body, further investigation was performed to see if it was also on the Müller cells as shown in our previous studies. Therefore, triple staining was performed on 21-day saline treated CNV retinas using anti-B_1_R (Figure 7A); GFAP (Figure 7B) and Kir4.1 antibodies, which is expressed strongly in Müller cells (Figure 7C). The merged microphotographs show the colocalization of B_1_R, GFAP and Kir4.1 (Figure 7D–F), confirming the expression of B_1_R in Müller cells.

### 3.5. R-954 Prevents Retinal Dysfunction

**Figure 8:**
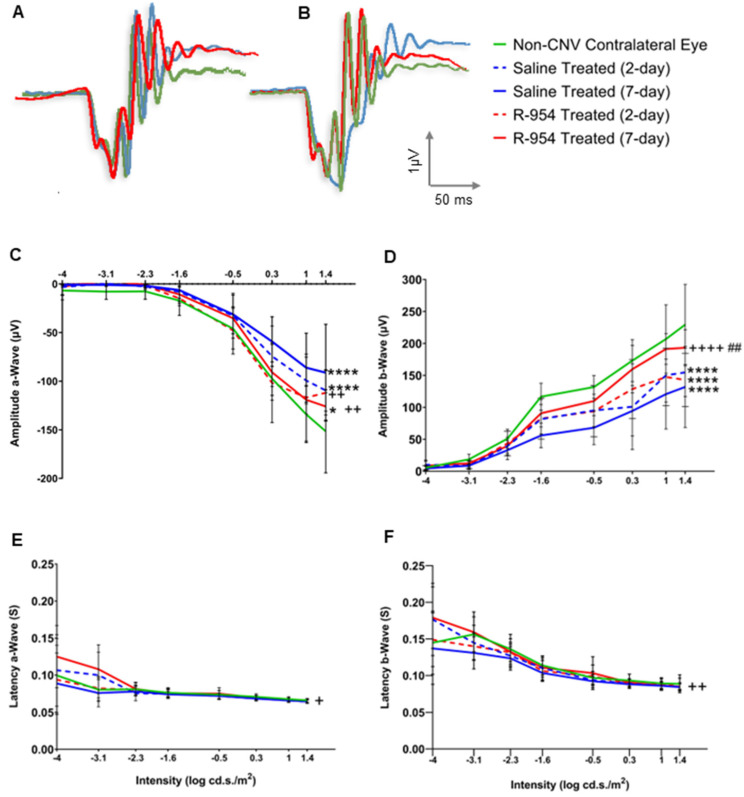
Visual function assessment by electroretinography (ERG) after laser induction of choroidal neovascularization (CNV) and treatment with R-954 in C57BL6 mice. Representative dark adapted ERG waveforms for a-wave and b-wave (**A**,**B**). Comparisons between R-954 and saline-treated mice were shown on a-wave amplitude (**C**) and latency (**E**) and b-wave amplitude (**D**) and latency (**F**) at day 2 and day 7 post-CNV. Data were presented as mean ± SD (n = 8 per group) and statistical analysis was performed using two-way ANOVA followed by Bonferroni test. Comparisons were made to non-CNV (*), saline (+) and between day 2 and day 7 of R-954 as indicated by (*^/+^ *p* < 0.02/0.04, ^++/##^ *p* < 0.001/0.002, ****^/++++^ *p* < 0.0001).

ERG amplitude and peak latency recorded in scotopic conditions demonstrated distinctive changes in the retinal functioning after CNV induction as compared to non-CNV contralateral eye in an intensity-dependent manner (*p* < 0.0001). Saline-treated CNV eyes show extensive decrease in the a- wave (−41 ± −16 and −34 ± −14 µV vs. −59 ± −21 µV) and b-wave (80 ± 20 and 64 ± 17 µV vs. 117 ± 30 µV) on day 2 and day 7 vs. non-CNV contralateral eye (*p* < 0.0001). In comparison, R-954 treatment retained close to normal amplitude values of a- (−49 ± −19 and −48 ± −20 µV) and b-wave (82 ± 20 and 101 ± 27 µV) on day 2 and day 7, respectively (*p* < 0.0001) (Figure 8C,D). In addition, there was a significant difference in the b-wave amplitude values between day 2 and day 7 of R-954 treated CNV eyes, indicating that recovery was greater on day 7 (*p* = 0.009) (Figure 8D). A significant increase in the ERG latencies of day 7 saline-treated CNV eyes; a-wave (*p* = 0.003) and b-wave (*p* = 0.0051) was observed compared to day 7 of R-954 -treated CNV eyes; however no significant difference was noted compared to non-CNV contralateral eye (Figure 8E,F). In addition, we also observed a significant decrease in the amplitude of sum of OPs in the saline treated CNV eyes in day 2 (155 ± 35 µV) and day 7 (89 ± 10 µV), compared to non-CNV eyes (350 ± 63 µV), whereas R-954 treated eyes retained normal OPs values in day 2 (277 ± 49 µV) and day 7 (352 ± 42 µV) (*p* < 0.0001).

## 4. Discussion

This study supports our previous findings that B_1_R is involved in inflammation and the development of CNV in neovascular AMD [16,17], and further highlights the salutary effect of the B_1_R antagonist R-954 in prevention of retinal degeneration and loss of retinal function in mice model of AMD. In the present study, we have further demonstrated that B_1_R remains upregulated until 21-day post-CNV and contributes to neo vessels formation. This information is relevant in terms of treatment strategy as the extension of CNV into the retinal or sub RPE space is considered an important sequela of AMD [26,27,28]. The preclinical mouse model simulates many of the features of neovascular human AMD, including the development of subretinal neovascular membranes, inflammation, and angiogenesis [5].

The formation of neo vessels and vascularized folds were clearly visible in the retina and choroids of 21-day saline-treated CNV eyes. The abundant CNV clusters and capillaries observed within the saline treated choroid were undoubtedly absent after R-954 treatment. R-954 treatment was also effective in reducing CNV area and volume. Even though there was no significant difference in the CNV area between 7-day and 21-day during R-954 treatment, a 4-fold significant reduction was detected in the CNV volume on 21-day as compared to 7-day. The present study also put emphasis on the possibility of CNV being a part of the spectrum of exudative AMD [29] that contributes to the upregulation of B_1_R in the retina, which further perpetuates the disease. Our results clearly show a growth of abnormal blood vessels causing leakage and damage in overlying retina in the CNV saline treated group. We assumed that there was a correspondence between the vascular abnormalities in the retina and the localization of the CNV spots on the choroids—although we did not evaluate it—but it is also probable that inflammatory factors might diffuse from the choroidal spots to different parts of the retina, contributing to spread damage. R-954 treatment was successful in preserving the normal retinal structure and vascular bed in all the treatment groups, suggesting that inflammatory factors are indeed responsible for the retinal damage.

One of the important characteristics of retinal degeneration is microglial activation and B_1_R has been shown to promote microglial proinflammation during CNV development [16,17]. Therefore, investigation of microglia expression was crucial for understanding the inhibitory activity of R-954. Earlier studies have shown the presence of microglial cells in the early stages of neovascular AMD due to their protective role in promoting the clearance of debris and apoptotic cells [30,31]. Similar effects of CNV was observed in the present study with the enhanced microglial expression in saline treated retina and choroid in the early (7-day) but also the late time frames (14-day and 21-day) of CNV development. The role of microglial cells in CNV development is complex and may depend on the specific context and stage of disease. Microglial cells can also contribute to neovascularization by producing pro-angiogenic factors such as IL-1β and TNF-α during later stages of the retinal CNV [32]. Importantly, these two cytokines are well known inducers of B_1_R expression [9,11].

Microglia are known to interact with other cells in the retina, such as endothelial cells and pericytes, to promote angiogenesis with ageing and display increased signs of gliosis [30,31,32,33]. Our results reveal the colocalization of B_1_R in the astrocytes/Müller cell population, but not on microglial cells (Iba-1 positive cells). Astrocyte/Müller cell end feet participates in the regulation of microvessels permeability (retinal-blood barrier) and are known to become active in response to injury [34]. Thus, B_1_R co-expression on glial end feet may have an impact on endothelial and vascular function, as shown previously in Alzheimer’s disease mice [11]. Previous studies have also shown a co-expression between GFAP and B_1_R in human AMD retinae [17] and in the preclinical rat model of AMD [16]. Moreover, an increase in the GFAP immunoreactivity is considered a hallmark of macro-gliosis [11] and gliosis increases vascular permeability and neovascularization in retina, thus exacerbating the progression of AMD [35]. Müller cells have similar roles as astrocytes [36], and GFAP expression in Müller cells is considered an indicator of tissue stress, and have been associated with the retinal degeneration [37]. This has been seen as an hallmark of B_1_R immunoreactivity in retinal cross-sections of the rat model of CNV [16], throughout the ganglion cell layer, inner nuclear layer, and outer nuclear layer of the retina, in retinopathies [9,15]. Our result in this study confirms a strong colocalization of B_1_R and GFAP expressing glial cells in the CNV retina. In addition, the intermediate filament protein Kir4.1 ubiquitously co-expressed in Müller cells of many mammalian species [38] also showed a clear co-expression with B_1_R.

Studies have revealed the contribution of increased number of microglia to the progression of the pathology and the degeneration of photoreceptors [30]. Hence, we investigated the beneficial impact of R-954 in monitoring retinal functional changes using scotopic ERG recordings. ERG is considered an effective strategy for the monitoring of function of different cell populations in the retina including photoreceptors and have been used to study changes in retinal function associated with the development and progression of neovascular AMD [39,40]. The results display a major decrease in the a-wave amplitude of the CNV mice whereas R-954 treated mice revealed normal ERG value, suggesting compromised photoreceptor cell function due to CNV development and inflammation can be prevented by B_1_R blockade. ERG a-wave amplitude reflects the electrical response of the photoreceptor cells (rods and cones) in the retina to a brief flash of light. The a-wave is the first negative deflection of the ERG waveform and reflects the initial depolarization of the photoreceptor cells in response to light. The amplitude of the a-wave is proportional to the number of functioning photoreceptor cells in the retina as well as to their sensitivity to light. In addition, we also observed a decrease in the amplitude of the scotopic b-wave, which reflects the function of bipolar cells in the retina [41]. The loss of photoreceptor cells can also lead to a reduction in the amplitude of the scotopic b-wave in ERG recordings, reflecting the function of bipolar cells in the retina, suggesting a loss of bipolar cell function during AMD. In addition, decrease in the amplitude of the sum of all Ops was observed after CNV induction, which reflects CNV effects on amacrine cells. This further suggests a loss of retinal function due to loss of amacrine cell function. The changes in ERG recordings observed in CNV mouse model are likely due to a combination of factors, including the direct effects of neovascularization on retinal cells, as well as the secondary effects of inflammation and oxidative stress associated with the disease. Several mechanisms have been proposed to explain the effects of AMD on photoreceptor and bipolar cell function such as oxidative stress and inflammation [42,43]. Our results show that treatment with R-954 has improved ERG recording in both a-wave and b-wave amplitudes suggesting the efficacy of R-954 to prevent the loss of photoreceptor and bipolar cell function. Significant improvement in the a-wave latency and b-wave latency was seen when comparison was made between day 7 of R-954 treatment and day 7 of saline treatment. Also, R-954 treatment seems to prevent the loss of amacrine cells as reflected with normal OPs within R-954 treatment group, suggesting again a beneficial impact of R-954 in prevention of retinal function during AMD. Hence B_1_R over expression is believed to play a deleterious role in the development and progression of AMD.

One of the main advantages of topical ocular anti-B_1_R therapy achieved with an antagonist is that it is safe and allows for self-administration on the surface of the eye. In recent years, intravitreal injections of antibodies inhibiting vascular endothelium growth factor (VEGF, strongly expressed by Müller cells and involved in neovascularization) have been commonly used to treat CNV [1,44], due to its predominant role on cellular proliferation, migration, and angiogenesis in AMD. The anti-VEGF aflibercept is currently the first line of treatment used in clinical practice [45,46]. The neovascularization regresses with anti-VEGF therapy and the loss of visual acuity stops. However, this therapy is not entirely safe, is highly expensive, and exhibits side effects on blood flow and the survival of neurons, as it also prevents the positive effects and vital influence of VEGF on photoreceptors and vessels [35,47,48]. Moreover, many AMD patients do not respond to anti-VEGF therapy or develop medical complications to it [1,5,35]. The B_1_R antagonist could thus be a valuable tool as an alternative approach for AMD treatment.

## 5. Conclusions

Our study provides compelling evidence that B_1_R antagonist treatment can inhibit inflammation, neovascularization, and angiogenesis in addition to restoring the photoreceptor and bipolar cell function in the retina of a preclinical mouse model of AMD. The restoration of retinal cell function needs to be further studied for the loss of behavioural, retinal, and cortical functions during progression of AMD. Our results also confirm the colocalization of B_1_R in astrocytic/Müller cell population and since the mechanisms underlying AMD are complex and multifactorial, further studies are needed to understand the interaction of B_1_R and astrocytic/Müller cell population during AMD. Our findings suggest that B_1_R may be one potential therapeutic target for neovascular AMD. Since the present study only determines the preventive role of R-954 antagonist, this study needs to be extended further to understand the curative role of R-954 in laser-induced AMD. In conclusion, we believe that R-954 treatment can be helpful to patients who do not respond to current anti-VEGF therapies.

## Data Availability

The data supporting the findings of this study are available within the article and upon reasonable demand.
